# Role of rhizosphere specific microbiome in enhancing soybean productivity across contrasting soil and crop management systems

**DOI:** 10.3389/fpls.2026.1830235

**Published:** 2026-05-21

**Authors:** Prem Ranjan, Diptimayhee Das, Vindhya Bundela, Aketi Ramesh, Rakesh Kumar Verma, Raghavendra Nargund, Urmila Manandhar, Rhae Drijber, Rakesh K. Upadhyay, Mahaveer Prasad Sharma

**Affiliations:** 1 Microbiology Section, Indian Council of Agricultural Research (ICAR)-National Soybean Research Institute, Indore, Madhya Pradesh, India; 2 Department of Agricultural Microbiology, Indira Gandhi Krishi Vishwavidyalaya, Raipur, Chhattisgarh, India; 3 Department of Agronomy and Horticulture, University of Nebraska-Lincoln, Lincoln, NE, United States; 4 Department of Natural Sciences, College of Arts and Sciences, Bowie State University, Bowie, MD, United States

**Keywords:** arbuscular mycorrhizal fungi, crop-soil management practices, microbiome, plant growth promoting rhizobacteria, rhizomicrobiome, soybean

## Abstract

Soybeans are a globally significant legume and oilseed crop with a diverse rhizospheric microbiome that can enhance sustainable agriculture by reducing the need for chemical fertilizers. These microbes can potentially impact plant growth and development through symbiotic (rhizobium and mycorrhizae) and non-symbiotic (plant growth-promoting rhizobacteria and fungi) interactions with soybean roots under optimal crop and soil management practices. During the production of soybeans, practices such as excessive use of fertilizers and pesticides, mono-cropping, and intensive tillage are often employed to achieve higher yields. However, these practices can alter the rhizomicrobiome communities and their interactions with soybean crops. Implementing optimal soil and crop management techniques can create a more favorable environment for rhizomicrobial communication with soybean roots, ultimately enhancing nutrient uptake for the soybean plants. In this review, we address how the soil rhizomicrobiome communicates with soybean roots, its role in promoting plant health and yield, and approaches to enhance soil rhizomicrobiome diversity and function through improved crop and soil management practices. Herein we synthesize current literature on soybean-microbe interactions, including both symbiotic and non-symbiotic relationships with an emphasis on how plant-microbe interactions within soybean cropping systems are influenced by agricultural practices such as crop rotation, intercropping, integrated nutrient management, and no-tillage. Greater understanding of the complexity underlying rhizosphere microbiome relationships will enable design of local cropping systems enhancing soybean yield along with improving soil health.

## Graphical Abstract

**Figure:**
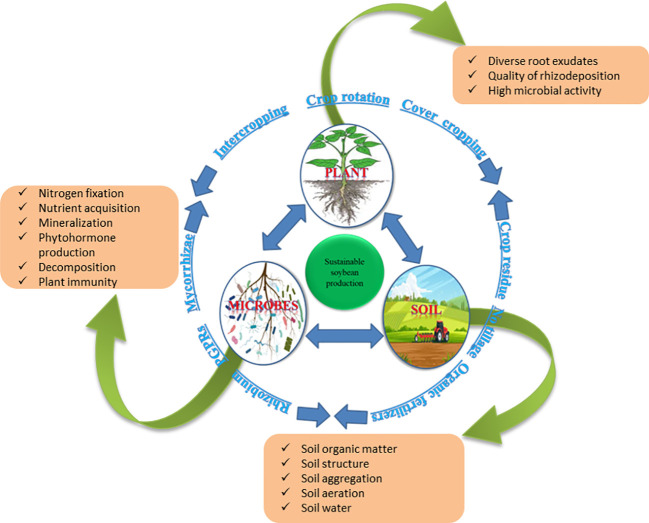

## 1 Introduction

Soybean (*Glycine max* L.) is a legume oil-seed crop cultivated worldwide and known for its high-quality protein (approximately 40-42%) and oil content (18-20%) (Clemente and Cahoon, 2009), which serves both human nutritional needs and livestock feed requirements (Montoya et al., 2017). As a result, soybean is not only a dietary staple crop but also a strategic crop in terms of economic stability and international trade (Sun et al., 2018). In developing economies, where soybean cultivation supports millions of smallholder farmers, its role in livelihood security and soil health management is particularly important. However, India’s average national yield remains relatively low, hovering around 1.2 t/ha, below the global average and has only modest incremental gains reported over the past two decades (Suresh et al., 2014; Raghavendra and Suresh, 2020).

The soybean crop faces numerous challenges, including drought, recurring flooding, soil degradation, and inadequate soil and crop management practices. Among these, poor crop and soil management is considered as a major contributor to the soybean yield loss (Egli and Hatfield, 2014; Sentelhas et al., 2015; de Souza Nóia and Sentelhas, 2020). Assessing potential yield and identifying yield gaps can offer valuable insights for enhancing crop productivity through improved soil and crop management practices (Silva et al., 2022).

Soybean, as a leguminous species, has the unique capacity to meet a significant part (60-80%) of its nitrogen requirements through biological nitrogen fixation (BNF), primarily mediated by symbioses with *Bradyrhizobium* spp (Bieranvand et al., 2003; Bharti et al., 2023; Santachiara et al., 2017). However, the efficiency of BNF largely depends on environmental conditions and management practices. Stress conditions such as soil acidity, compaction, water deficits, and nutrient imbalances can inhibit rhizobial activity and nodulation efficiency, thereby increasing dependency on exogenous nitrogen inputs (King and Purcell, 2001; Salvagiotti, 2008). While added nitrogen can marginally improve yields in high-performing or stressed environments, indiscriminate fertilizer use has been linked to declines in soil enzymatic activities (e.g., nitrogenase, urease), microbial diversity, and ecological function (Dar and Bhat, 2020; Bharathi et al., 2024). These findings support the growing consensus that sustainable productivity cannot rely solely on chemical inputs but must integrate biological and ecological processes (Silva et al., 2022; Bebber and Richards, 2022).

Recent meta-analyzes and global reviews emphasize the importance of systems-based approaches that integrate soil biological health with agronomic optimization to narrow the soybean yield gap (Fischer, 2022; Zhong and Zhong, 2024). Key components of such strategies include crop rotation, conservation tillage, targeted nutrient management, and organic matter amendments. Crop rotation especially involving cereals like maize or wheat has been shown to enhance soil microbial diversity, disrupt pest and pathogen cycles, and improve nutrient cycling efficiency, leading to consistent yield benefits over monoculture systems (Mourtzinis et al., 2017; Yuan et al., 2022; Chamberlain et al., 2021). Likewise, conservation tillage practices such as no-till or reduced-till systems preserve soil structure, enhance water retention, and reduce carbon losses, while creating favorable microhabitats for beneficial soil biota (Halwani et al., 2019; Silva et al., 2023).

A crucial, yet often underutilized, component of sustainable soybean intensification is the soil rhizomicrobiome, a highly dynamic microbial community associated with the rhizosphere that coordinates nutrient transformations, stress tolerance, and plant immune modulation (del Carmen Orozco-Mosqueda et al., 2018; Mahmud et al., 2021; Zhang et al., 2026). The rhizomicrobiome comprises a diverse array of bacteria, archaea, fungi, and actinomycetes, with key functional groups such as nitrogen-fixing rhizobia, phosphate-solubilizing bacteria, plant growth-promoting rhizobacteria (PGPR), and arbuscular mycorrhizal fungi (AMF) playing pivotal roles in plant-microbe interactions (Kumawat et al., 2022; Costa et al., 2024; Sarao et al., 2025). These microbial taxa are not only responsible for direct plant benefits such as improved nutrient acquisition and growth regulation via phytohormones and growth molecules, but also for indirect effects including pathogen suppression and abiotic stress mitigation. The structure and function of the rhizomicrobiome are strongly influenced by human management of soils and crops. Evidence from long-term field trials and recent reviews demonstrates that practices such as organic matter addition, reduced tillage, cover cropping, and microbial inoculants can significantly enhance microbial diversity and resilience, leading to improved crop performance and ecological stability (Simarmata et al., 2019; Moretti et al., 2024). For instance, conservation agriculture systems in diverse agroecosystems have been shown to support keystone microbial taxa and facilitate beneficial symbioses that are otherwise reduced under intensive mono-cropping and high-input regimes (Thierfelder et al., 2016; Kochiieru et al., 2020).

Keeping the above into consideration, harnessing the soil rhizomicrobiome potential offers a promising approach to align agronomic and ecological goals in soybean production. Integrating microbial-centric practices into existing cropping systems can enhance nutrient-use efficiency, enhance stress tolerance, and improve yield sustainability-outcomes that are critical under the dual pressures of climate change and food security. This review synthesizes current knowledge on how diverse crop and soil management practices influence the rhizomicrobiome and explores their potential in sustaining soybean productivity across varying agro-ecological contexts.

## 2 Assessment of the soybean rhizomicrobiome

The rhizosphere, the narrow zone of soil surrounding plant roots serves as a natural habitat for various microorganisms that collectively form the rhizomicrobiome. This microbiome plays a significant role in sustaining soil health, plants, and enhancing resilience (Jamil et al., 2022; Pandey and Saharan, 2025). Soybean, one of the most widely cultivated legume crops, has a unique rhizomicrobiome that is influenced by its symbiotic relationships with nitrogen-fixing bacteria. This interaction enhances soil health, increases crop yields, and improves disease resistance along with N fertilizer source (Moretti et al., 2018; Gouda et al., 2018; Naorem et al., 2026). Unlike many other crops, soybean establishes a highly efficient symbiotic relationship with specific strains of *Bradyrhizobium*, including *Bradyrhizobium japonicum* (Sidorin et al., 2026), *Bradyrhizobium diazoefficiens* (Bender et al., 2022b; Knoll and Mirza, 2026), and *Bradyrhizobium elkanii* (Lu et al., 2025). This relationship allows soybean to fix atmospheric nitrogen thereby reducing dependence on synthetic fertilizers that can, over time, negatively impact long-term agricultural and environmental sustainability and soil health (Hungria and Mendes, 2015; Peoples et al., 2021; Moretti et al., 2018; Bender et al., 2022a).

In contrast to soybean, other legumes, such as the common bean, have shown a limited capacity for biological nitrogen fixation (BNF) and often require the addition of nitrogen fertilizers for optimal growth (Hungria and Mendes, 2015; Peoples et al., 2021; Moretti et al., 2018). This limited ability to fix nitrogen has been largely attributed to the host plant’s high promiscuity, as it tends to associate with a variety of native rhizobial strains that are well adapted to the local soil environment (Graham, 1981). Worldwide, according to Peoples et al. (2021), soybean alone derives about 66% of its nitrogen from the atmosphere compared to ~ 60% for all pulses combined. When compared to cereals such as maize or wheat, the soybean rhizosphere harbors a distinct microbial community with a higher proportion of nitrogen-fixing bacteria, capable of deriving up to 90% of their nitrogen from atmosphere (Rajguru et al., 2024). The signaling network among plant roots and their rhizomicrobiome is influenced by abiotic stress and soil properties, which can change the composition of signaling molecules and rhizomicrobial communities (Han et al., 2023; Qu et al., 2023). Previous studies have shown that abiotic stressors such as drought, extreme temperatures, and salinity can alter the composition and quantity of root exudates. These changes in exudates can affect the diversity and structure of rhizospheric communities, helping the plant improve its tolerance to stress. For example, soybean plants treated with a bacterial consortium of *Bradyrhizobium* spp. and *Azospirillum brasilense*, along with the application of microbial secondary metabolites, demonstrated improved nodulation, growth development, grain yield, and enhanced tolerance to oxidative damage during dry spells (Moretti et al., 2020). Additionally, rhizobacteria that produce the extracellular enzyme 1-aminocyclopropane-1-carboxylate deaminase can help mitigate the adverse effects of heat and water deficiency by reducing ethylene concentration (McMillan et al., 2022).

The rhizosphere of soybean also hosts beneficial bacteria such as *Bacillus* and *Azospirillum* which increase in abundance throughout the growing season and play crucial roles in soybean cultivation. For instance, *Azospirillum* enhances root growth (Cassan et al., 2026), while *Bacillus* are well studied for disease suppression (Luqman et al., 2025) and together support sustainable soybean cultivation in tropical regions (Prando et al., 2014; Mun et al., 2024; Villalobos et al., 2022). A recent study carried out by Moretti et al. (2024) based on metagenomic analysis showed that a bacterial consortium composed of *Bradyrhizobium japonicum*, *Bradyrhizobium diazoefficiens*, *Azospirillum brasilense*, and *Bacillus subtilis* has significantly influenced the soybean holobiont, where both the rhizomicrobiome and the fertility of the rhizosphere soil has enhanced. Moreover, synthetic bacterial communities have been shown to promote communication between rhizobia and their host plants, thereby strengthening the symbiotic effectiveness of this mutually beneficial system (Han et al., 2020). Microbial signaling molecules, such as Lipo-chitooligosaccharides (LCOs), that regulate symbioses tend to increase in the rhizosphere over time and are essential for root nodule formation (Martins et al., 2026; Scherer et al., 2026).

The composition of the soybean rhizomicrobiome changes dynamically throughout developmental stages, especially during nodulation, and influences overall microbial diversity (Wang et al., 2020a; Tamrakar et al., 2026; Xie et al., 2026). Management practices efficiency, reducing the need for nitrogen fertilizers in legume-cereal intercropping systems (Cheng et al., 2023). Additionally, soybean crop residues left after harvest can also help enrich nitrogen levels in the soil (Kumar and Babalad, 2018). Moreover, differences in rhizosphere bacterial communities have been observed between high-yielding and low-yielding regions (Yuan et al., 2023). Network analysis has identified key microbial taxa such as Anaerolineae, Micromonosporaceae, Planctomycetes, Nitrospira, and Rhizobium that significantly influenced soybean yields (Niraula et al., 2022). These findings highlight the potential for managing rhizosphere microbiomes to enhance soybean productivity, particularly in marginal areas across diverse agro-ecological systems.

## 3 Rhizosphere: a microbial world

**Figure 1:**
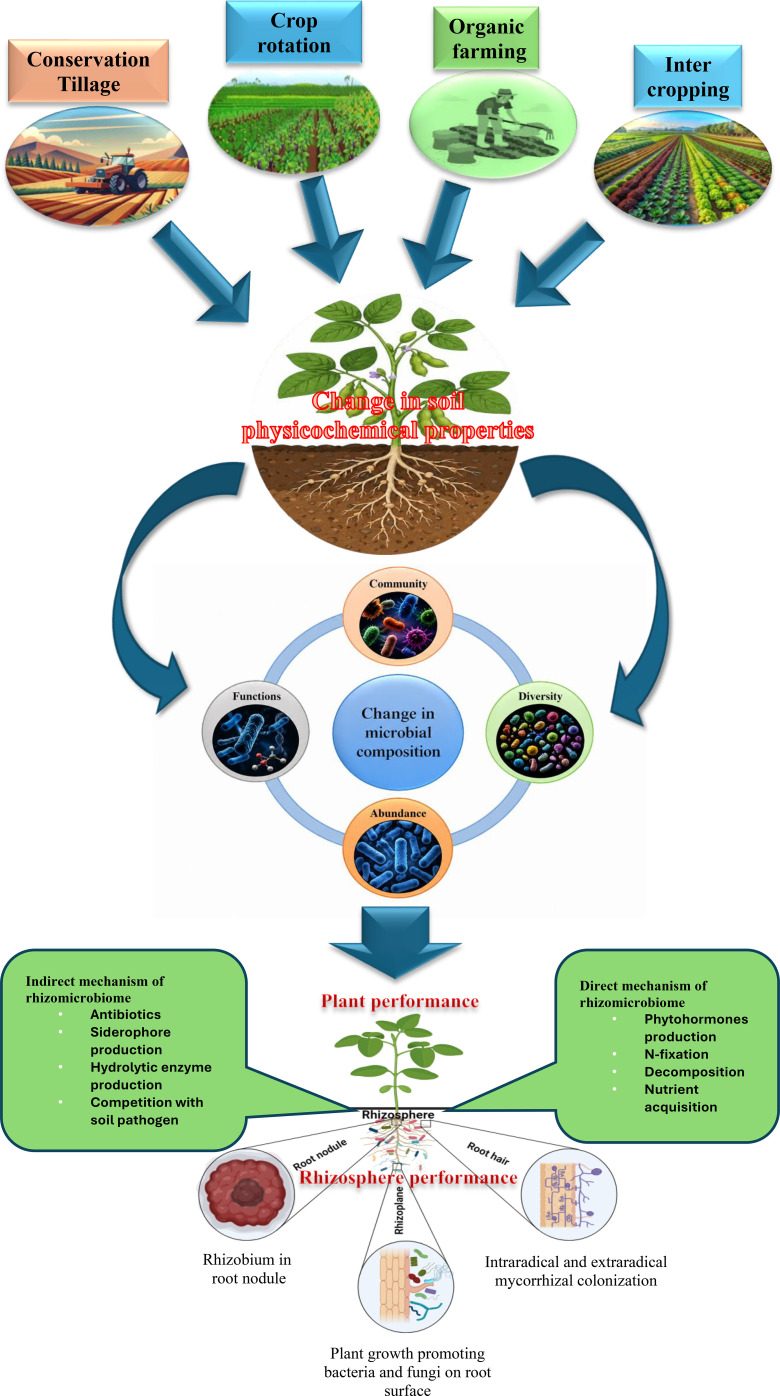
Illustrates the interactive effects of soil and crop management practices, such as crop rotation, conservation tillage, organic farming, and intercropping, on the physicochemical properties of soil. These practices alter the composition of the soil rhizomicrobial community in the soybean rhizosphere, which enhances both plant and rhizosphere performance through rhizomicrobial mechanisms.’.

The rhizosphere is home to large and diverse community of microorganisms (Hiltner, 1904) termed the ‘rhizomicrobiome’ and includes bacteria, fungi, protista, nematodes, viruses, and algae (Kumar et al., 2020; Meena et al., 2017) (**Figure 1****).** It is a dynamic zone around the root where root exudates, soil microbes and habitat constraints drive soil chemical and biological processes (Prasad et al., 2019; Hiremath et al., 2024). Thus, the rhizosphere, is a nutrient-rich zone that functions as a hub for nutrient exchange among microbes, soil, and plants (Bashir et al., 2016; Fahde et al., 2023). Within this dynamic environment, unique components such as the root epidermis (rhizoplane), adhering soil, and rhizosheath (root hair) contribute to nutrient uptake, and exchange of carbon dioxide (CO_2_) and oxygen (O_2_). Consequently, this zone becomes the hub of plant-microbe-microbe interactions that influence soil biochemical cycling and plant productivity (York et al., 2016; Thepbandit and Athinuwat, 2024). The characteristics of the rhizosphere may depend on plant species’ root structure and exudation patterns through alterations in microbial diversity and their activity (Kuzyakov and Razavi, 2019). For example, exudation of aspartic acid through soybean roots shows a strong association with *Glomus* species of AM fungi in soybean-maize intercropping systems (Zhang et al., 2024). Similarly, the flavonoid-mediated exudation patterns in legume-cereal cropping systems predominantly facilitate rhizobium symbiosis with legume crops. Similarly, attraction of soil bacteria by plant root volatiles is also evaluated by several studies (Schulz-Bohm et al., 2018) including involvement of ethylene with soil-specific microbial species near growing roots (Chen et al., 2020). These exudation patterns not only promote the enrichment of rhizobial colonization but also enhance the presence of nitrogen-fixing rhizomicrobes beyond rhizobia, such as *Pseudomonas*, which improves nitrogen assimilation and nodulation in legume plants (Qiao et al., 2024). When compared to bulk soil, the rhizosphere harbors relatively higher microbial populations, containing up to 1 billion bacteria, 200 million fungi, and about 10 thousand protists per gram of soil (Sokol et al., 2022). In spite of high microbial density in rhizosphere, Mendes et al. (2014) reported that the assembly of the soil microbiome in the soybean rhizosphere is less diverse compared to that of bulk soil. This is because rhizosphere communities are a subset of the bulk soil reservoir. The preferential selection of the soybean rhizomicrobiome from bulk soil depends on functional cores related to the metabolism of nitrogen, iron, phosphorus, and potassium in soybean plant. These functions contribute to the benefits for the soybean plant, such as growth promotion and enhanced nutrition. Therefore, the rhizosphere is an ideal habitat for specific microbial diversity related to specific metabolic pathway, where soil acts as a medium and martin (Mendes et al., 2011; Martins et al., 2026). Thus the concept of the rhizomicrobiome encompasses the diverse microbial activities that are crucial for maintaining soil health, enhancing soybean productivity, sustaining environmental remediation, and global climate regulation (Lynch, 2024; Kumar and Chhajer, 2026).

## 4 Plant-microbe interactions in the rhizosphere

Signaling from plant to soil microbes via plant-secreted molecules has been shown to facilitate beneficial interactions (Mhlongo et al., 2018; de Andrade et al., 2023). Most research focuses on signaling between plants and symbionts within the rhizomicrobiome, particularly those involving rhizobium (Roy et al., 2020; Ma et al., 2026; Yuan et al., 2026) and mycorrhizae (Hassan and Mathesius, 2012; Tian et al., 2026). Legume crops use chemical attractants such as flavonoids to attract LCO secreting rhizobia and initiation of root nodules (Poole et al., 2018; Olah et al., 2005). Similarly, plants secrete strigolactones to attract arbuscular mycorrhizal fungi (AMF), which subsequently produce LCOs to facilitate root colonization and nutrient exchange (Aliche et al., 2020; Phour et al., 2020). Under non-symbiotic conditions, some soil microbes (and plants) produce iron chelating substances (e.g. siderophores) that facilitate plant iron uptake under iron-stress conditions (Martinez-Medina et al., 2017; Bundela et al., 2026). Similarly, an endophytic consortium of *Bacillus* sp. and *Pseudomonas* sp. Can enhance phosphate solubilization efficiency in wheat cultivars grown in phosphorus-deficient soil (Martin et al., 2017) and some free-living rhizobacteria may fix atmospheric nitrogen for non-legumes (Jacoby et al., 2017; Dahiya et al., 2026).

Microbes communicate both within and between species primarily by producing autoinducers or sensors, a process known as quorum sensing (Waters and Bassler, 2005). These auto-inducers enable microbes within communities to control cell density and manage behavioral changes in microbial species, which may benefit plant health (Venturi and Keel, 2016). Among, quorum-sensing compounds, N-acyl homoserine lactones (AHLs), have been extensively studied (Papenfort and Bassler, 2016). Scherlach and Hertweck (2018) have reported that many substances, including trehalose, glucose, oxalic acid, and thiamine, serve as signaling molecules between rhizobacteria and fungi. For example, mycorrhizal helper bacteria (e.g. *Pseudomonas fluorescens*), release thiamine into the rhizosphere to promote AM fungal hyphal growth while ectomycorrhizal fungi (e.g. *Laccaria bicolor*) produce trehalose, which acts as a chemoattractant for the mycorrhizae helper bacteria and other plant growth-promoting rhizobacteria (Hassani et al., 2018).

## 5 The role of rhizomicrobiome in improving plant health

### 5.1 Nutrient acquisition

**Figure 2:**
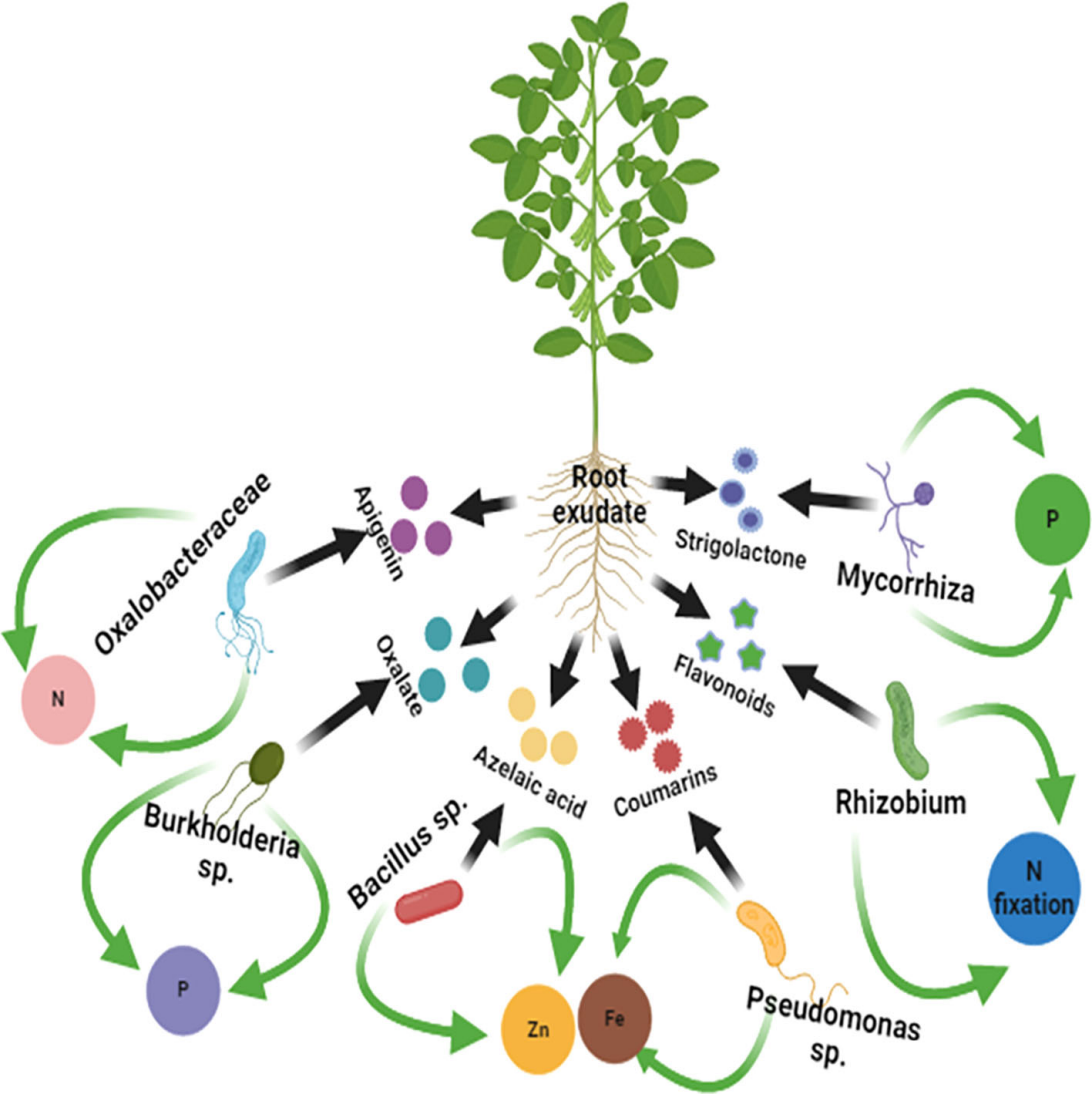
Elucidates the interactions between soybean plants and their rhizomicrobiome, driven by root exudates. The roots of the plants release a diverse array of exudates, including organic acids (oxalate and azelaic acid), flavonoids, coumarins, and signaling molecules such as strigolactones. These compounds selectively recruit beneficial microbial taxa such as *Rhizobium* for biological nitrogen fixation, *Pseudomonas* and *Bacillus* spp. for nutrient solubilization and phytohormone production, *Burkholderia* spp. for plant growth promotion, and oxalobacteraceae for organic acid metabolism.

The plant rhizomicrobiome plays a crucial role in enhancing plant nutrient acquisition (**Figure 2**). The benefits of mycorrhizal symbioses and *Rhizobium* for nutrient acquisition, particularly for phosphorus uptake, have been extensively studied (Dellagi et al., 2020). AM fungi increase the surface area of roots, facilitating enhanced nutrient absorption and transfer of minerals and nutrients from soil to plants. In comparison, under symbiotic conditions, rhizobacteria fix atmospheric nitrogen by transforming molecular nitrogen into ammonia, which legume plants can utilize as a nitrogen source (Gaby and Buckley, 2011). Other members of the rhizomicrobiome such as *Azospirillum* in maize (Garcia de Salamone et al., 1996) and wheat (Boddey et al., 1986) enhance nutrient availability by altering root architecture to enable roots to capture more soil nutrients and water (Tao et al., 2019). Plant growth-promoting rhizobacteria (PGPR) also transform, solubilize, and mineralize insoluble iron and inorganic phosphate, making them more accessible to plants. According to Ahmed and Holmstrom (2014), these microorganisms secrete low molecular weight organic compounds with high-affinity iron chelators responsible for enhancing iron uptake from the soil. In iron deficient soil, siderophores improve the reduction of iron into more soluble forms that can be absorbed by plant roots (Pecoraro et al., 2021). Some strains of *Pseudomonas, Rhizobium, Bradyrhizobium, Alcaligenes*, and *Serratia* showed high potential for producing siderophores, thereby facilitating the availability of iron (Fe^3+^) for plants and thus plant growth (Verma et al., 2019).

### 5.2 Phytohormone production

The role of plant hormones in plant-microbe symbioses has been studied (Foo et al., 2019). Auxin, a crucial plant hormone in plant-microbe interactions, is synthesized by most soil microbes (Luo et al., 2018). Plant growth-promoting rhizobacteria (PGPR), for instance, can modify the phytohormone pool and enhance its production (Grover et al., 2021). Auxin is mainly synthesized from tryptophan, particularly indole-3-acetic acid (IAA) which promotes root differentiation, root hairs thereby increases root exudation. IAA also influences plant pigmentation, photosynthesis, metabolite synthesis, and responses to light, gravity, and flowering (Nazir et al., 2018). Certain aerobic sporogenous bacteria produce ACC-deaminase, which helps in regulation of ethylene production (Kudoyarova et al., 2019). Some strains of *Bacillus* such as *B. licheniformis* and *B. pumilus* stimulate plant growth via gibberellin production (Verma et al., 2019). Gibberellins play essential roles in transportation of metabolites, chloroplast formation, cell division, stem morphogenesis, and leaf senescence (Rizza and Jones, 2019).

### 5.3 Plant immunity

The rhizomicrobiome also improves plant growth through inhibition of plant pathogens through various defense mechanisms (Hossain et al., 2017). These mechanisms include mycoparasitism, induced systemic resistance, and systemic acquired resistance, but also by outcompeting pathogens for nutrients and space. For example, the effect of resident rhizomicrobiome-based immunity on plants can happen through induced systemic resistance (ISR) where members of the rhizomicrobiome community stimulate the plant immune system to develop resistance against phytopathogens (Fitzpatrick et al., 2020). Most rhizobacteria produce secondary metabolites that inhibit the growth of soil-borne pathogens and their activities while some fungi can release antibiotics against soil pathogens (Geetanjali and Jain, 2016). Various studies have found that the synthesis of antimicrobial substances such as benzoxazinoids (Hu et al., 2018), coumarin (Stringlis et al., 2018), and lytic enzymes (e.g. protease, chitinase, and β-1,3 glucanase), as well as pathogen-inhibiting volatile compounds, may enhance disease resistance against pathogens (Naznin et al., 2014). Certain strains of *Trichoderma* fungi also produce secondary metabolites such as polyketide, harzianic, heptelidic acid, terpines, gliotoxin, peptaibols, gliviorin, massoilactone and gliosoprnins which reduce soil pathogens (Khan et al., 2020).

### 5.4 Improving the soil structure, aggregation, and health

Soil aggregates are the fundamental units of soil structure formed by binding soil particles and organic matter into stable aggregates that influence organic carbon dynamics and biodiversity in soil. These aggregates, comprised of organic matter, soil minerals, water and gases provide microhabitats for soil microbes and other living organisms (Canton et al., 2009). Aggregates conserve soil organic carbon by creating some physical barriers between microbes and their substrates thereby limiting enzymatic activity and the decomposition process. In turn, soil microbes positively affect soil structure formation by incorporating organic matter and minerals into soil aggregates. Through this a diversity of microbial species with relationship of plant, maintains the balance between carbon sources and sinks (Pellegrino et al., 2022).

Soil organic matter is the largest reservoir of organic carbon and nitrogen in the soil ecosystem. It originates from the decomposition of animal and plant residues and associated microbial activities (Liang et al., 2017). The decomposition of organic matter involves both the biochemical degradation of complex organic compounds into simpler organic and inorganic compounds and the physical disintegration of the substrates. This process, often called “microbial decomposition” which is central to energy transfer and nutrient cycling within the soil ecosystem, releasing essential macro and micronutrients (Lehmann and Kleber, 2015). Consequently, the movement of organic material and its breakdown modulates nutrient and energy transfer within and across soil ecosystems (Smith et al., 2012).

## 6 Key drivers of soil rhizomicrobiome community composition and diversity

Effective agricultural management practices account for the physical, chemical, and biological qualities of soil composition. Among these, soil organisms and their functions are one of the most important and sensitive indicators of soil and plant health quality. Research studies have shown that various sustainable agricultural practices such as tillage intensity, organic amendments, mulching, residue management, crop rotation, use of cover crops and intercropping influence the composition and diversity of the soil microbial community. Additionally, anthropogenic factors such as land use changes and pollution can also impact soil microbial diversity (Kuffner et al., 2004) (**Table 1**).

**Table 1:** Contribution of soybean rhizosphere microbiome on crop productivity and microbial soil health under different soil and crop management practices.

Dominant members of rhizomicrobiome community	Management practices	Plant-soil-microbial interaction effects	Reference
*Bacillus, Mycobacterium, Streptomyces and Sphingomonas*	No-tillage and integrated crop-livestock	Higher plant growth-promoting rhizobacteria in integrated crop-livestock (33.8%) as compared to no-tillage (26.5%)	Costa et al. (2024)
*Bradyrhizobium japonicum;Bacillus megaterium; Azotobacter chroococcum*	Fertilizer management with rhizomicrobiome inoculation	The highest effectiveness was recorded in seed yield (6-25%), protein yield (0.3-29%), oil yield (2-26%), protein content (1-4%), and oil content (0.4-3%) compared to control.	Miljakovic et al. (2024)
*Bacillus* sp.*, Serratia marcensces*	Cover crop + soybean	The soybean yield increased by 22% and phosphorus content in the soil was 95% higher compared to the control.	Silva et al. (2024)
*Globisporangium, Pythium, Wilsoniana*	Soybean-Maize-Wheat rotation + no tillage-Maize-Wheat rotation -Maize-Wheat rotation+ no tillage	Enhanced soybean seedling by 21% which was positively correlated with soybean yield under no-tillage practices.	Gahagan et al. (2023)
*Bradyrhizobium sp*	Soybean + Maize intercropping	Efficiency of nitrogen fixation increased by 69% under intercropped soybean compared with mono-cropped soybean	Cheng et al. (2023)
*Gemmatimonas,Gaiella, Mortierella, Guehomyces*	Soybean-maize rotation system	Increased soybean yield by 11.27% compared with continuous soybean cropping and strongly positive correlate with soil organic carbon	Sun et al. (2023)
*Aneurinibacillus, Shingoaurantiacus, Solibacillus, Cohnella, Tumebacillus*	No-tillage+Soybean-maize rotation+ inoculation of *Urochloa brizantha*	Firmicutes (39%) and Actinobacteria (29%) were abundant in soils from soybean-maize management systems under conservation tillage	Araujo et al. (2023)
*Burkholderia, Caballeronia, Paraburkholderia, Sphingomonas, Mortierella*	Soybean-Maize rotation	The soybean yield and available phosphorus in soil were 36.78% and 23.24% higher under crop rotation compared to the continuous cropping system	Sun et al. (2022)
*Gaiella, Bacillus, Microlunatus, and Bradyrhizobium*	Soybean-corn rotation	Soybean yield was significantly higher (9.11%) under long-term crop rotation	Li et al. (2022)
*Bradyrhizobium, Ensifer and Desulfovibrio*	Inoculation of mycorrhiza with continuous cropping system	AM fungi inoculation increased the plant height by 7.99%, Bradyrhizobium by 23.58%, and total bacterial abundance by 47.36% compared to non-inoculated soybean plants	Jie et al. (2021)
*Paludibaculum, Nordella, Candidatus solibacter, Chujaibacter, Rhodanobacter*	Soybean-Corn rotation+ No tillage + no-tillage	Increased soil organic matter from 4.6% to 5.4% under proper crop and soil management practices	Behnke et al. (2021)
*Bradyrhizobium, Gemmatimonas, Schizothecium Cellulomonas, Rhodomicrobium, Pyrenochaetopsis*,	Soybean-Corn rotation	Improve soybean yield from 4719 to 5614 kg per ha, soil protein from 5613 to 7360 mg per kg of soil and β-glucosidase from 0.93 to 1.19 mg p-nitrophenol per gm	Neupane et al. (2021)
*Bradyrhizobium* sp.*, Sphingomonas, Gaiellales, Streptomyces*	Soybean no-tillage + organic management	Soybean growth enhanced by 11.3% (roots) and 30.1% (stems) at R2 stage under proper management practices	Reid et al. (2020)

### 6.1 Plant diversity and agricultural practices driving the composition of the rhizosphere microbiome

The soil microbiome exists in the rhizosphere, which is the area of soil surrounding plant roots and is home to a diverse range of microorganisms (Park et al., 2023). Variation in microbial community composition is influenced by plant type and traits (Martins et al., 2024). Plants have a genetically encoded ability to change the composition and diversity of the rhizomicrobiome, which is influenced by plant-derived resources such as root exudate (Schlatter et al., 2015; Qiao et al., 2017; Yang et al., 2021). Crop rotation significantly changes the structure of bacterial and fungal communities in the soil by supplying nutrients through different types of root exudates and plant residues (Hartmann and Six, 2023). Increasing the diversity of crop species from two to three enhances the impact of crop rotation on both bacterial Shannon diversity and species richness. This improvement may occur because two-species rotations usually involve highly contrasting crops, such as legumes and non-legumes, which create significant changes in the conditions of the rhizosphere due to variation in root exudate and specific microbial associations linked to each crop (Lu et al., 2024). Legumes have the ability to fix atmospheric nitrogen through their symbiotic relationship with rhizobia, enriching the soil with bioavailable nitrogen forms, such as ammonium and nitrate (Li et al., 2023). When legumes are replaced by non-leguminous plants, these plants rely more heavily on soil nitrogen, which alters bacterial competition and selection pressures in the soil (Hibbing et al., 2010). This change tends to favor bacterial taxa that are involved in nitrogen mineralization and decomposition, leading to increased diversity and structural changes within the microbial community (Company et al., 2022; Ling et al., 2022). Moreover, legumes are known to release root exudates that are rich in flavonoids and phenolics. These compounds can selectively enhance certain bacterial taxa in the rhizosphere (Leoni et al., 2021), particularly by promoting the growth of symbiotic and beneficial bacteria such as *Rhizobium* and *Pseudomonas* (Qiu et al., 2024; Wu et al., 2025). Therefore, the reduction of these compounds when transitioning from legumes to non-legumes may lead to more significant restructuring of bacterial communities, compared to the opposite transition, where symbiotic nitrogen-fixing bacteria gradually regain dominance (Leoni et al., 2021). The introduction of diverse substrates through legume-non-legume rotations creates broader ecological niches, which in turn foster greater bacterial α-diversity (Sexton et al., 2017; Fang et al., 2024) and fungal β-diversity (Tedersoo et al., 2014). The increased β-diversity of fungal communities and the shifts in fungal structure that occur with the transition from legume to non-legume crop types are likely due to disruptions in fungal symbioses. Many legume crops are significantly associated with AM fungi, which improve phosphorus uptake and help shape fungal communities (Li et al., 2009). When legumes are replaced with non-legumes, these mycorrhizal-dependent networks can be disrupted, leading to an increase in β-diversity as saprotrophic and pathogenic fungi become more prevalent (Scheublin et al., 2004; Van Der Heijden et al., 2015). Additionally, differences in litter input and root architecture between legumes and non-legumes further influence soil microhabitats and the availability of resources for fungal communities. This can promote the emergence of new fungal taxa and result in greater spatial heterogeneity (Pioli et al., 2020). Many soil bacterial species (Sieber et al., 2021) and fungal species (e.g., mycorrhiza and endophytic fungi) (Mesny et al., 2023) have been observed to co-evolve with specific host plants and display strong preferences for them. Consequently, introducing different crops can reshape rhizosphere soil microbial communities, leading to distinct interactions between crops and soil microbes (Berg and Smalla, 2009). This host-specific selection probably plays a significant role in the enhanced bacterial and fungal diversity observed in crop rotation systems.

### 6.2 Tillage practices influencing rhizosphere microbiome composition

Tillage practices alter the physical, chemical, and biological properties of soil causing a significant effect on the health and productivity of agricultural ecosystems. Conventional tillage, while traditionally used for weed control and seedbed preparation, has severe drawbacks such as loss of soil nutrients and organic matter, reduced biochemical activity, and increased soil erosion. Therefore, these effects can negatively impact soil biodiversity, which is considered as one of the biggest environmental threats to sustainable agriculture; thus, such negative effects must be avoided (Gajda et al., 2017; Angon et al., 2023). Conservation tillage practices, especially no-tillage with residue retention, are essential for influencing the diversity and structure of microbial communities (Wang et al., 2020b; Khan et al., 2023). The relationship between tillage and soil bacterial communities is complex, as tillage influences the soil’s physicochemical properties and is interconnected with soil microbial communities (Trivedi et al., 2017). These methods minimize soil disturbance, promote the accumulation of organic matter, and establish a more stable microenvironment for soil microbial proliferation. Compared to conventional practices, nutrient-rich soils under conservation tillage practices better support the growth of copiotrophic microorganisms by modifying the structure and co-occurrence patterns of the diazotrophic community (Li et al., 2021). Alternative tillage practices such as minimum tillage and zero tillage can improve soil quality and conserve arbuscular mycorrhizal fungi (Wilkes et al., 2021) reduce erosion (He et al., 2011; Kovács et al., 2023) and soil diversity (Engell et al., 2022; Domnariu et al., 2025). Agnihotri et al. (2021) and Liang et al. (2025) showed that a long-term, zero tillage, cover crop (ZT-CC) system was more effective for enhancing carbon sequestration, partially due to increased AMF biomass and glomalin production. Long-term conservation tillage decreases the alpha diversity of the diazotrophic community in the soil profile and significantly affects community structure. However, it increases the overall density of both bacterial and diazotrophic communities. This difference may be linked to the increased relative abundances of Proteobacteria and *Bradyrhizobium* observed in conventional tillage practices (Ling et al., 2025). Previous studies have shown that Alphaproteobacteria and Betaproteobacteria, which are the dominant classes within Proteobacteria, are typically classified as copiotrophic bacteria (Trivedi et al., 2013). These dominant copiotrophic soil organisms may thrive due to the nutrient resources derived from returning crop residues, which promote their growth (Fierer et al., 2007; Fan et al., 2019). Proteobacteria are a diverse group of bacteria that play crucial roles in nitrogen cycling, particularly in processes such as nitrification and denitrification. Studies have shown that this phylum is more abundant in soils with lower levels of disturbance under conservation tillage (Galazka et al., 2017; Zhang et al., 2022). In contrast, Planctomycetes thrive in soils with low organic carbon availability, which can lead to an increase in the accumulation of recalcitrant carbon in the soils (Bei et al., 2018). This improvement in carbon accumulation is also suggested by a decrease in peroxidase activity under conservation practices. The dominance of these two major groups of microorganisms in soil underscores their important roles in enhancing nutrient availability, facilitating the breakdown of organic matter, and maintaining soil fertility. This highlights their significant contributions to carbon and nitrogen cycling (Tang et al., 2019; Luan et al., 2020). Similarly, according to Santellanez-Arreola et al. (2025), at the genus level, the genera *Pseudarthrobacter*, *Blastococcus*, and *Rhizobium* were found to be more abundant in conventional tillage treatments compared to conservation agriculture. This increased presence of these genera in conventional tillage suggests that the soil management practices associated with this method may enhance their growth. Conversely, the genera *Mesorhizobium*, *Gemmata*, *Amaricoccus*, *Pedomicrobium*, and *Bradyrhizobium* were more prevalent in soils managed under long-term conservation agriculture practices. Understanding these microbial patterns can assist farmers and researchers in evaluating the effects of various tillage and residue management practices on microbial communities.

### 6.3 Organic farming and rhizosphere microbiome composition

Organic farming is gaining popularity in today’s agriculture due to the negative impacts of synthetic fertilizers on soil health and the environment. Organic agriculture practices conserves soil chemical and biological properties such as nutrient mineralization, nutrient availability, higher organic matter and abundance of soil microbiota (Schrama et al., 2018; Dai et al., 2025) and sustains ecological services (Philip Robertson et al., 2014) through the use of plant and animal-derived eco-friendly fertilizers that also provide optimum organic matter to the soil (Lori et al., 2017; Ma et al., 2025). Organic and chemical fertilization have contrasting effects on soil microbial diversity and structure (Saharan et al., 2023). Organic fertilization, which involves materials derived from plants or animals, such as compost or manure, adds organic matter and improves soil health (Krause et al., 2023). It has been shown to enhance microbial diversity, increase enzyme activity, and improve soil physicochemical properties. For example, one study found that organic cultivation increased the abundance of dominant bacterial phyla, such as Proteobacteria and Acidobacteria, as well as fungal phyla like Ascomycota and Basidiomycota (Xu et al., 2022). In contrast, while chemical fertilization provides concentrated nutrients that can boost crop yields, prolonged use may lead to reduced microbial diversity (Liang et al., 2019; Sun and Badgley, 2019). Long-term application of chemical fertilizers has been shown to reduce microbial diversity while increasing the presence of copiotrophic taxa, such as Actinobacteria and Proteobacteria (Chu et al., 2007; Hartmann et al., 2014). Paradoxically, chemical fertilizers also lead to a rise in pathogenic fungi, which worsens soil-borne diseases. In contrast, using chemical fertilizers alongside manure has demonstrated synergistic benefits by stabilizing microbial communities and enhancing nutrient use efficiency (Ding et al., 2016; Hu et al., 2022). However, soil microbial communities are primarily influenced by a select group of “keystone taxa,” such as Cyanobacteria and Glomeromycota, rather than by dominant high-abundance groups like Proteobacteria and Ascomycota (Chen et al., 2018).

### 6.4 Intercropping and rhizosphere microbiome composition

Intercropping is an effective crop management practice that enhances soil and plant health (Singh et al., 2016; Yu et al., 2025) by increasing nutrient use efficiency, optimizing land resource utilization, and reducing pest attacks (Mousavi and Eskandari, 2011; Bo et al., 2025; Raza et al., 2025). Growing legumes with non-legume crops is a basic principle of intercropping in which, there is assumed no competition between intercropped species. The legumes fix atmospheric nitrogen to fulfil the nitrogen requirement of non-legume crops, thereby improving overall nitrogen use efficiency of the intercrop system (Xu et al., 2020; Jing et al., 2025). Such practices promote microbial diversity, higher yields (Tang et al., 2020; Wang et al., 2025) and beneficial rhizomicrobiome interactions that support soil biological processes.

In intercropping systems, crops influence soil microenvironments by competing for nutrients through root exudates, which in turn affects soil microbial diversity (Wang et al., 2018). Compared to the long-term monoculture of soybeans, soybean-maize intercropping enhances the diversity of rhizosphere microorganisms and improves the complexity and stability of their interaction networks (Chang et al., 2022). Notably, intercropping has distinct effects on the diversity and structure of bacterial and fungal communities (Xiao et al., 2023). For example, soybean-maize intercropping increases both bacterial and fungal diversity, modifies the soil community structure through interspecific interactions, and boosts the relative abundance of beneficial bacterial taxa that play a role in nutrient cycling (Guo et al., 2024). In soybean-based intercropping systems, the increased diversity of rhizosphere microorganisms has been linked to enhanced root exudation (Yong et al., 2012). Additionally, when soybean is intercropped with maize, it releases greater amounts of daidzein and genistein, which help reshape the structure and diversity of the rhizobia community in the rhizosphere. As a result, nodule nitrogen fixation and overall yield are improved (Lin et al., 2023).

## 7 Conclusion and future prospects

Currently, farmers rely heavily on chemical fertilizers, insecticides, and pesticides for crop production. Sustainable agriculture faces the challenge of meeting crop demands while minimizing environmental impact of excessive chemical fertilizer use. Conventional practices such as mono-cropping, intensive tillage, and indiscriminate fertilizer use disrupt beneficial plant-soil interactions. Effective soil and crop management practices significantly influence the rhizomicrobiome, which is crucial for plant nutrient uptake and soil health. Thus, future research in sustainable soybean production should focus on enhancing rhizomicrobiome diversity and function through adoption of crop rotation, intercropping, integrated nutrient management, use of cover crop and conservation tillage practices. These practices should replace existing conventional practices, to harness the native pool of soil nutrient levels and microbial communities for sustaining crop yields.

Over all, this review highlights the importance of rhizomicrobiome diversity for sustainable soybean production. Enhancing rhizomicrobiome diversity advocates an eco-friendly alternative to reduce reliance on synthetic fertilizers and pesticides, promoting plant-microbe interactions, improving soil fertility, and supporting plant health. To maintain soil diversity and foster these interactions, practices such as crop rotation, intercropping, no-till farming, and organic amendments are essential for a resilient soil ecosystem. This review offers insights into advancing sustainable soybean production through effective soil and crop management practices.
